# Supplementary material for: The mitochondrial pyruvate carrier regulates adipose glucose partitioning in female mice
Source: Mol Metab. 2024 Aug 11;88:102005. doi: 10.1016/j.molmet.2024.102005 (PMC11382204; doi:10.1016/j.molmet.2024.102005)

**SUPPLEMENTAL FIGURE LEGENDS**

**Supplemental Figure 1 (related to Figure 1): siRNA mediated depletion of MPC1 in 3T3L1 adipocytes**

Representative western blot and normalized, quantified protein expression of the mitochondrial carrier protein 1 (MPC1) in differentiated 3T3L1 adipocytes following reverse transfection with MPC1 siRNA. Cells were reverse transfected at 5 days post-differentiation as described in materials and methods. MPC1 protein expression was quantified in cell lysates at 2-, 3-, 5-, and 8-days post-transfection to determine the optimal time for subsequent assays in transfected cells (shown in Figure 1E). (**C)** mRNA expression of selected genes in 3T3L1 adipocytes treated with DMSO vehicle (VEH) or 5μM UK5099 throughout differentiation, normalized to 18S control and presented relative to VEH day 0.

**Supplemental Figure 2 (related to Figure 2):** **Metabolic phenotype of human cohorts**

(**A**) Blood glucose, (**B**) plasma insulin, (**C**) plasma free fatty acid concentrations during an oral glucose tolerance test, and **(D)** clinical and metabolic parameters in female and male human subjects with normal glucose tolerance (NGT) or with combined impaired fasting glucose and impaired glucose tolerance (IFG/IGT). *P<0.05, **P<0.01 for NGT vs IFG/IGT. Data are n=7 in each group.

**Supplemental Figure 3 (related to Figure 3):** **Further characterization of adipose explants from *Mpc1*^AD-/-^ mice**

(**A**) *Ex vivo* incorporation of [2-^14^C] pyruvate into the fatty acyl moieties of total lipids in inguinal adipose explants from male and female *Mpc1*^AD-/-^ mice or LoxP^+/+^ controls. (**B - E**) Rates of glycerol release (**B**), and non-esterified fatty acid release (**D**, **E**), and calculated non-esterified fatty acid re-esterification (**C**) from epididymal (EAT) or inguinal (IAT) adipose explants from male *Mpc1*^AD-/-^ mice or LoxP^+/+^ controls under basal (unstimulated) conditions or during treatment with insulin or forskolin plus triacsin C. *P<0.05, **P<0.01 for *Mpc1*^AD-/-^ vs LoxP^+/+^. Data are mean ± SE for at least five mice per group.

**Supplemental Figure 4 (related to Figure 4):** **Further characterization of lipogenic gene expression and liver contribution**

(**A - B**) mRNA expression of lipogenic genes in inguinal adipose tissue iWAT, (**C**) *in vivo* incorporation of intraperitoneally administered [U-^14^C] glucose into the fatty acyl moieties of total lipids (lipogenesis), (**D**) mRNA expression of lipogenic genes and **(E)** mRNA expression of lipid oxidation genes in livers from male and female *Mpc1*^AD-/-^ mice or LoxP^+/+^ controls fed either a high fat western-style diet (WD) or a zero fat, sucrose enriched diet (ZFD) for 24 weeks. ^P<0.05 for female vs male. Data are mean ± SE for 8-10 mice per group. **(F)** *GYK* mRNA expression in human adipose tissue from patients characterized in Figure 2.

**Supplemental Figure 5 (related to Figure 8):** **Further characterization of *Mpc1*^AD-/-^ mice under dietary stress**

(**A** - **B**) Absolute body weight and (**C** – **D**) blood glucose concentrations during an oral glucose tolerance test for male and female *Mpc1*^AD-/-^ mice or LoxP^+/+^ controls fed either a zero fat, sucrose enriched diet (ZFD **A - B**) or a high fat western-style diet (WD **C - D**) for 24 weeks. **P<0.01 for *Mpc1*^AD-/-^ vs LoxP^+/+^. Data are mean ± SE for 8-10 mice per group.


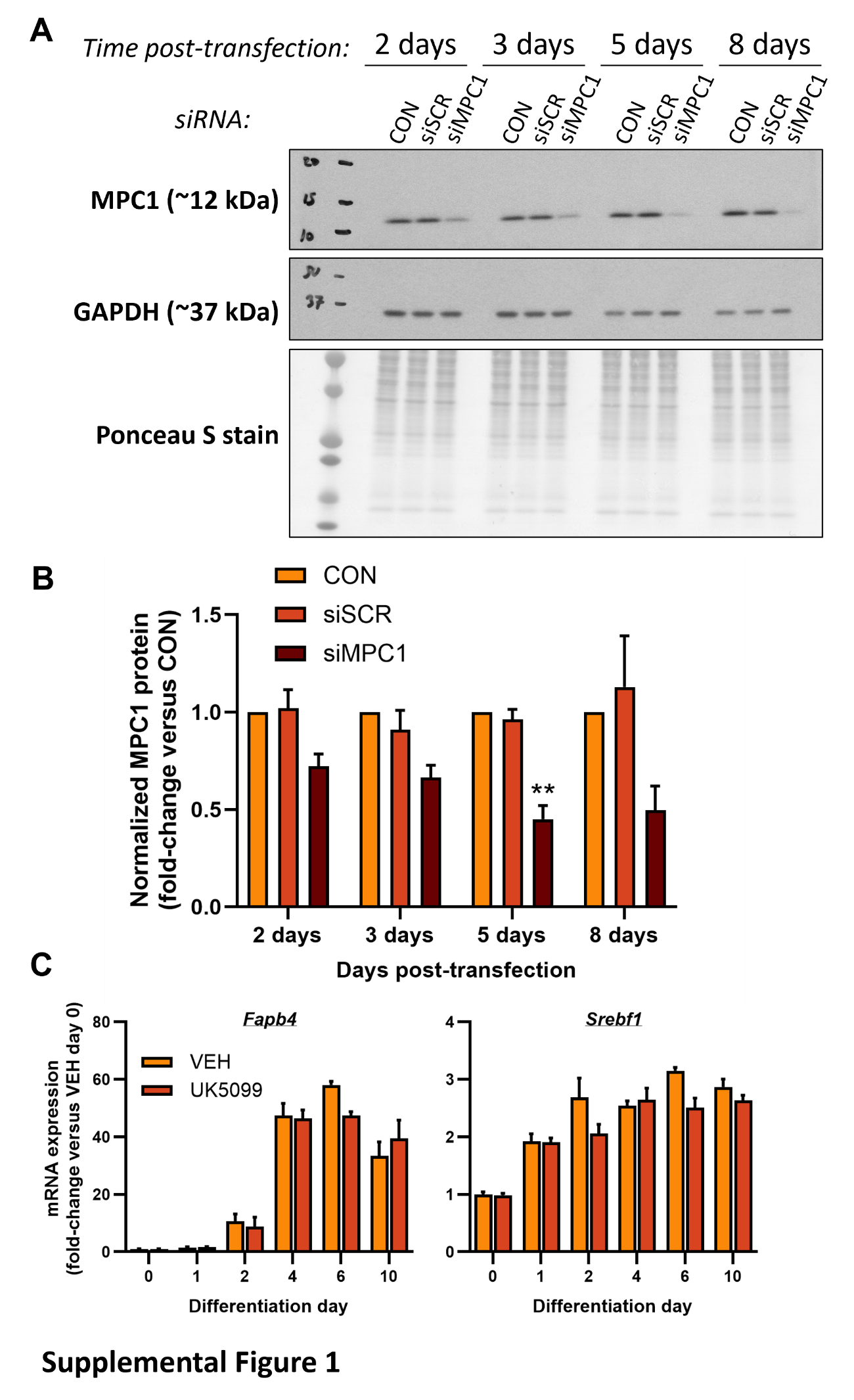

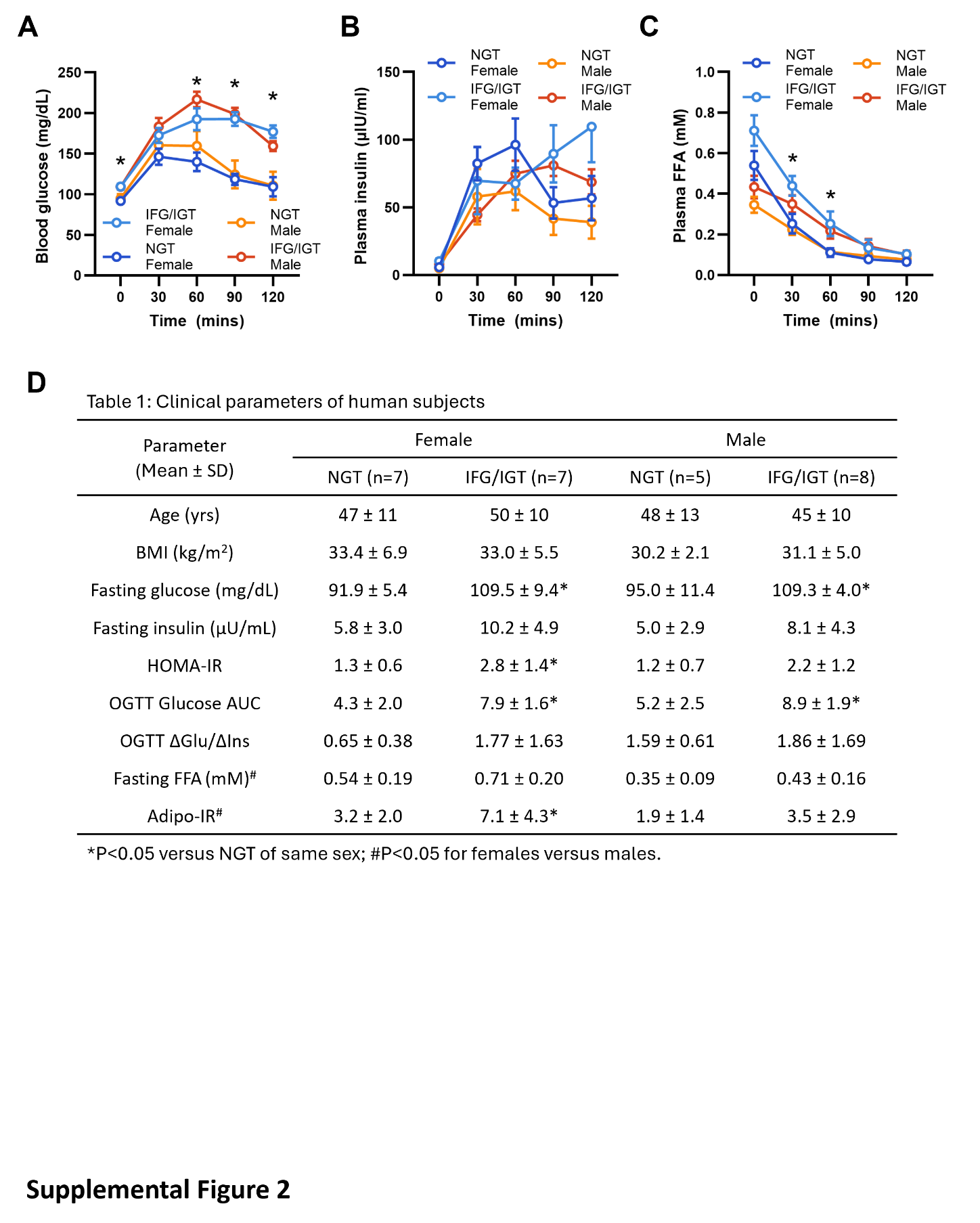

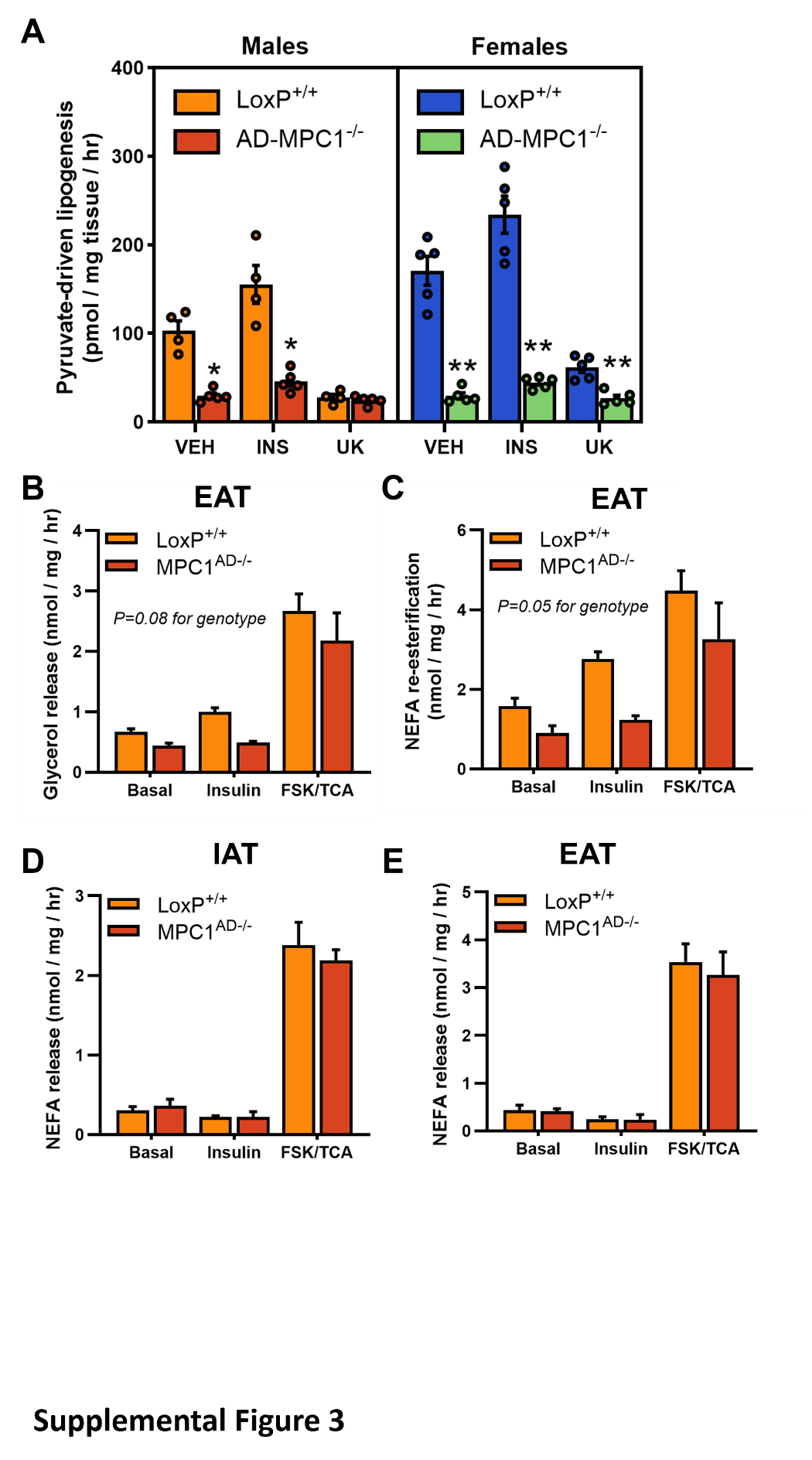

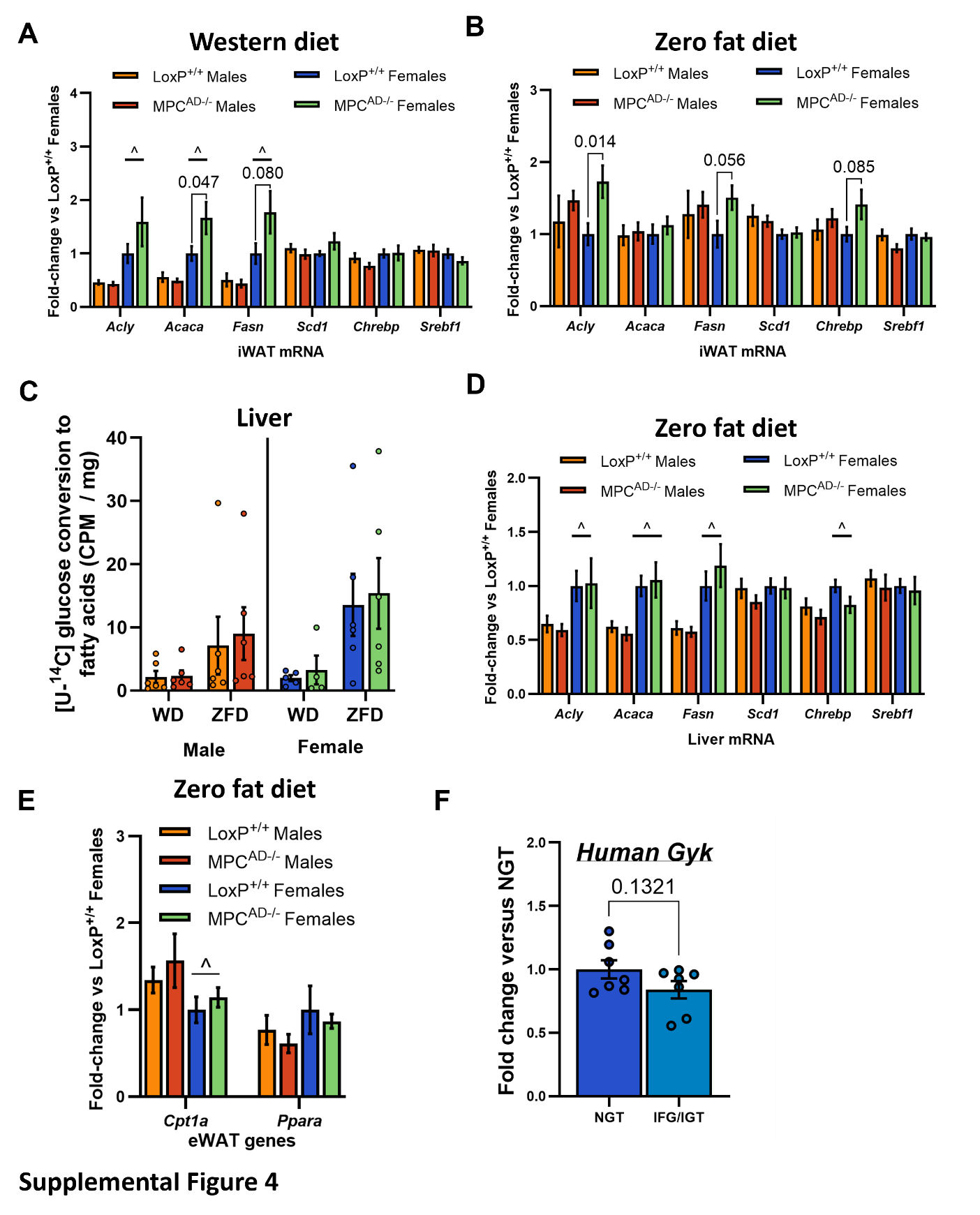

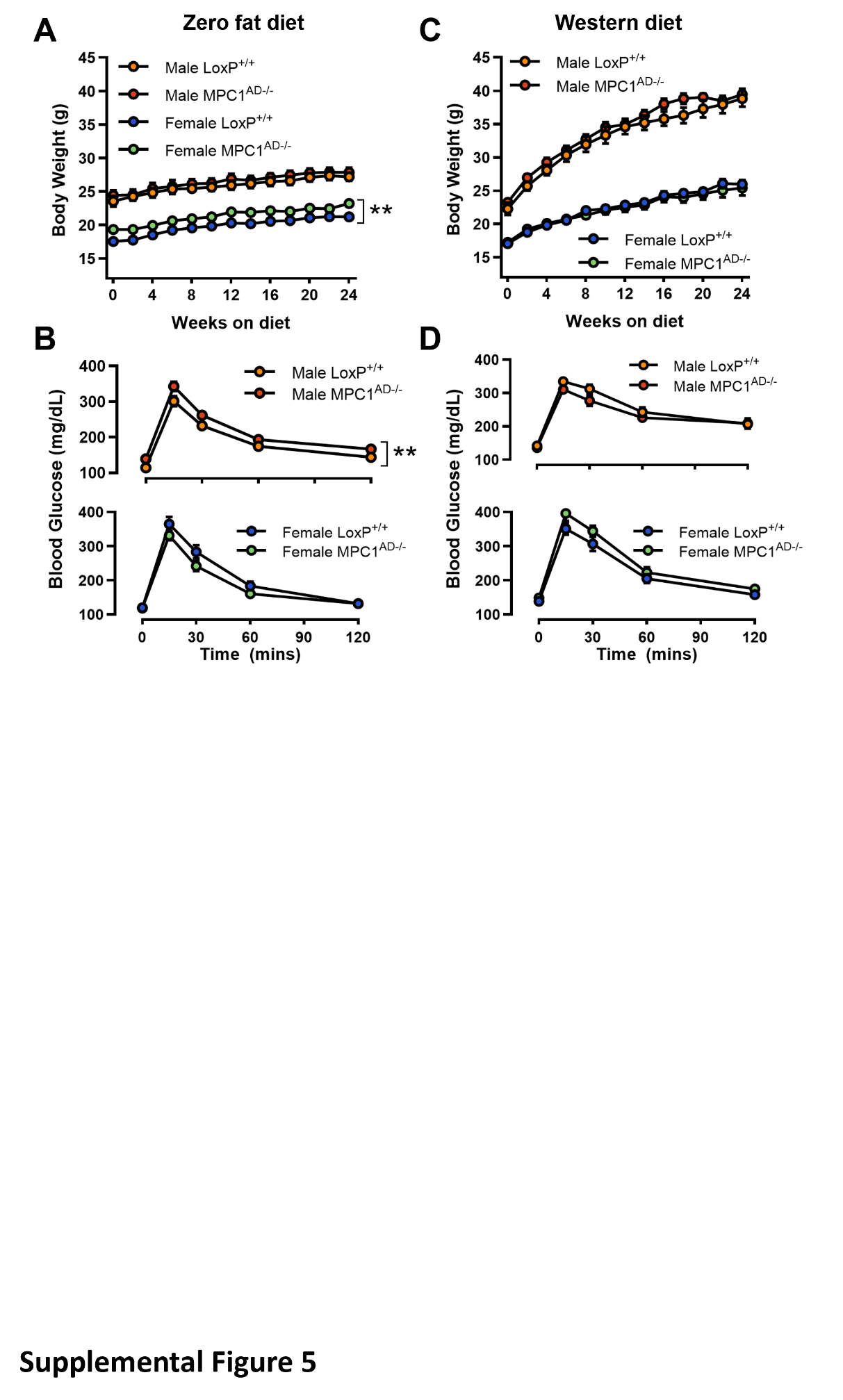

Supplement: Multimedia component 1 [file mmc1.docx]
